# Zero confirmed cases: The ways we curb COVID-19 in Taiwanese prisons

**DOI:** 10.7189/jogh.10.020377

**Published:** 2020-12

**Authors:** Kah Kheng Goh, Mong-Liang Lu, Susyan Jou

**Affiliations:** 1 Department of Psychiatry, Wan Fang Hospital, Taipei Medical University, Taipei, Taiwan; 2 Department of Psychiatry, School of Medicine, College of Medicine, Taipei Medical University, Taipei, Taiwan; 3 Graduate School of Criminology, National Taipei University, Taipei, Taiwan

**Figure:**
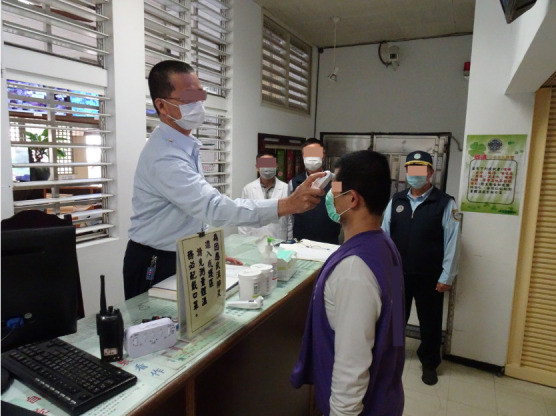
Photo: All prisoners, prison staffs, and visitors are required to check body temperature and asked to wear the surgical masks whenever in contact with others (from Ministry of Justice, used with permission).

The problem of transmission of infectious diseases in prison has come into focus amid the COVID-19 pandemic. Even outwith the immediate COVID-19 pandemic, it is well known that prisoners are at higher risk of infectious diseases than those in the wider community at other times [1]. It has been reported that most prisoners return to their communities with their illness occasionally untreated and sometimes worsened. These prisoners increase the public health burden by acting as reservoirs of infection [1]. It needs to be fully acknowledged that prison health is an issue of public health. Prison COVID-19 outbreaks have been reported and documented in China, India, Iran, Egypt, Malaysia, USA, Italy, UK, Congo, Nairobi, Salvador, Brazil, Colombia – indeed, almost every country where people in the wider community have contracted COVID-19 [2].

## DECARCERATION POLICY DURING COVID-19

Overcrowding and inadequate health services within the prison estate have fueled the prison as a highly infectious environment [3]. National level data for the majority of all countries worldwide indicate have prison occupancy levels exceeding their officially reported capacity [4]. Accessibility to health service, testing capacity, the supply of personal protective equipment are budget-constrained and are not being prioritized for inmates [5]. Calls for decarcerating (early release for example) prisoners convicted of low-level crimes and misdemeanors during this pandemic has been encouraged by the UN High Commissioner for Human Rights [6]. As a solution to mitigate the harms of COVID-19 outbreak in prison [7], several countries including Iran, France, Italy, Child, USA, and Indonesia have taken action to reduce the prison population by releasing “low-risk” offenders. Besides a general decarceration policy, prison-specific guidance for responding to COVID-19 has also been introduced [8].

## HOW TAIWANESE PRISONS RESPOND TO COVID-19

With its proximity to China, Taiwan was expected to have the second-highest number of COVID-19 cases [9]. There are approximately 60 000 inmates in Taiwanese prisons, with 115% prison occupancy level and the cell spatial density is only 2.3 m^2^ per person. Most of the prisoners occupy a single large cell, with approximately 16 to 20 inmates per cell. Since the outbreak of COVID-19 until 6 June, zero cases have been reported in Taiwanese prisons. The prisoners followed the same principles of the COVID-19 testing as for those in the wider community, which was around 1274 tests per confirmed case, considered adequate testing in Taiwan.

Due to concern about risk management in the community, no decarceration of prisoners has taken place in Taiwan. However, the Taiwanese government has adopted several proactive measures to minimize the possibility of a COVID-19 health catastrophe developing in the prisons. Measures have included the establishment of the joint planning and central command with Central Epidemic Command Center (CECC), the prevention, control, and risk management among the prisoners, the cooperation and involvement of prison staffs, and the environment sanitizing.

### Joint planning and central command with Central Epidemic Command Center (CECC)

A first crucial step was creation of a rapid response to COVID-19 through the establishment of CECC as an operational command point to integrate resources of the administration, the academic, medical, and private sectors. CECC enables direct communication among central and local authorities, including the prison and correctional authorities in developing an overall response to the crisis. The Guidelines for Prevention and Control of COVID-19 was developed by CECC. The Agency of Corrections, Ministry of Justice was adopted and introduced its own guideline that applied to all prisons. At least one response hospital each in six special municipalities was designated to be response for isolation of the patients with Emerging Infectious Diseases before this pandemic and the numbers of response and isolation hospitals were increased to 134 during COVID-19 pandemic. The suspicious cases in the prisons were first reported to CECC and transferred to those response hospitals that assigned by CECC, for screening, testing, and further management. Updated information and resources were thus provided daily to local prisons, including educational materials, screening and testing tools, and other equipment. The government made appropriate requisition for surgical masks, thermometers, and other resources which ensured the prisons were equipped with adequate resources.

### Prevention, control, and risk management

Weekly, all the prisoners were equipped with two surgical masks by the authorities. Prisoners were asked to wear the surgical masks whenever in contact with others. Good personal hygiene habits were implemented by all prisoners, especially during mealtimes and use of toilet facilities. All the prisoners are required to check body temperature twice a day and whenever they left and re-entered the prison for court hearing, work release, or other conditions. For those who re-entered the prisons or after their daily routine classes, they were mandated to bathe and change their clothes before entering their own cells. Unlike other countries, inmates in Taiwanese prisons have a highly structured activities program, filled with intensive activities; thus, they had a relatively low chance of close individual contact. Arguably, this acted as a form of social distancing, and this could also be a factor leading lower risk in the COVID-19 outbreak. Although no additional formal limitations were put on prisoner entry or re-re-entry from prison to the community, nevertheless, prisoners were encouraged to avoid unnecessary risk of infection. Prisoners were encouraged to use the medical clinics in prisons and reduce the community hospital visits. At least 2 medical clinics were available daily in each prison, including specialist clinics that operated weekly or monthly depending on the demand. The robustness of Taiwan’s health care systems arguably enables the prison health system to maintain its continuing everyday function during this pandemic.

Following, the criteria announced by the CECC, those who displayed symptoms of fever, cough, other acute respiratory symptoms, an abnormal sense of smell or diarrhea and with history of travel, or close contact with anyone with those symptoms were tested for COVID-19. To avoid transmission and spread within prisons, isolation areas were designated for suspicious cases. Prisoners who had body temperature ≥37.5°C or with upper respiratory tract symptoms were being quarantined in the isolation areas for 14 days. New prisoner entrants were asked to stay at the isolation areas for 14 days before being allocated to a prison cell. Authorities were asked to check these new entrants’ travel and contact history by using their National Health Insurance card data accessed via the online “MediCloud System”.

While seeking to maintain a level of isolation, Taiwanese authorities also recognized the importance of human contact for prisoners during this pandemic. Visits were not prohibited, except for volunteering and educational visits. All prison visitors were required to have body temperature checks, wear surgical masks, and report their travel history, occupational history, and contact history. Those who failed to comply were allowed only telephone visits.

### Cooperation and involvement of prison staff

All prison staff were trained on basic COVID-19 disease knowledge, hand hygiene practice, and use of appropriate prevention protective equipment. They were asked to ensure self-health monitoring, including regularly checking body temperature and the wearing of a surgical mask. Prison staffs who developed fever, cough, abnormal sense of smell, diarrhea or other acute respiratory symptoms and who had a past 14-day travel history or close contact with anyone with symptoms, were asked to report it to CECC and isolate at home for 14 days. Local health agencies contacted those self-isolating at home to check their health twice a day. All prison staffs were required to be tested before returning to work.

### Environment sanitizing

All visitors were asked to use 75% alcohol sanitizer for disinfection. Environment sanitizing was done after every visit session. All the equipment, including clothing and coverlets used in isolation areas was disinfected before being removed from the cells.

## CONCLUSIONS

The COVID-19 crisis has mobilized jurisdictions to release prisoners in many countries to minimize the risk of COVID-19 outbreak in prison. Early release of prisoners, particularly when the resources are scarce, may pose a threat to public safety. It is clear that there are no easy answers. Taiwanese prisons are among the most overcrowded prisons in the world. Yet, rather than a simple decarceration policy, the authorities in Taiwan chose to fight the COVID-19 pandemic proactively through evidently effective and well implemented strategies. There may be lessons for other countries in considering the detail of Taiwan’s success.
